# In Vitro Transcriptome Analysis of Cobalt Boride Nanoparticles on Human Pulmonary Alveolar Cells

**DOI:** 10.3390/ma15238683

**Published:** 2022-12-06

**Authors:** Mehmet Enes Arslan, Arzu Tatar, Özge Çağlar Yıldırım, İrfan Oğuz Şahin, Ozlem Ozdemir, Erdal Sonmez, Ahmet Hacımuftuoglu, Metin Acikyildiz, Fatime Geyikoğlu, Adil Mardinoğlu, Hasan Türkez

**Affiliations:** 1 Department of Molecular Biology and Genetics, Faculty of Science, Erzurum Technical University, Erzurum 25050, Turkey; 2 Department of Otorhinolaryngology, Faculty of Medicine, Ataturk University, Erzurum 25240, Turkey; 3 Department of Pediatrics, Pediatric Cardiology, Faculty of Medicine, Ondokuz Mayıs University, Samsun 55139, Turkey; 4 Advanced Materials Research Laboratory, Department of Nanoscience & Nanoengineering, Graduate School of Natural and Applied Sciences, Ataturk University, Erzurum 25240, Turkey; 5 Department of Medical Pharmacology, Medical Faculty, Atatürk University, Erzurum 25240, Turkey; 6 Department of Chemistry, Faculty of Science and Art, Kilis 7 Aralık University, Kilis 79000, Turkey; 7 Department of Biology, Faculty of Arts and Sciences, Atatürk University, Erzurum 25240, Turkey; 8 Science for Life Laboratory, KTH-Royal Institute of Technology, SE-17121 Stockholm, Sweden; 9 Centre for Host-Microbiome Interactions, Faculty of Dentistry, Oral & Craniofacial Sciences, King’s College London, London SE1 9RT, UK; 10 Department of Medical Biology, Faculty of Medicine, Atatürk University, Erzurum 25240, Turkey

**Keywords:** cobalt boride nanoparticles, toxicogenomics, human pulmonary alveolar epithelial cells, pathway analysis, in vitro, cytotoxicity

## Abstract

Nanobiotechnology influences many different areas, including the medical, food, energy, clothing, and cosmetics industries. Considering the wide usage of nanomaterials, it is necessary to investigate the toxicity potentials of specific nanosized molecules. Boron-containing nanoparticles (NPs) are attracting much interest from scientists due to their unique physicochemical properties. However, there is limited information concerning the toxicity of boron-containing NPs, including cobalt boride (Co_2_B) NPs. Therefore, in this study, Co_2_B NPs were characterized using X-ray crystallography (XRD), transmission electron microscope (TEM), scanning electron microscope (SEM), and energy-dispersive X-ray spectroscopy (EDX) techniques. Then, we performed 3-(4,5-dimethyl-thiazol-2-yl) 2,5-diphenyltetrazolium bromide (MTT), lactate dehydrogenase (LDH) release, and neutral red (NR) assays for assessing cell viability against Co_2_B NP exposure on cultured human pulmonary alveolar epithelial cells (HPAEpiC). In addition, whole-genome microarray analysis was carried out to reveal the global gene expression differentiation of HPAEpiC cells after Co_2_B NP application. The cell viability tests unveiled an IC50 value for Co_2_B NPs of 310.353 mg/L. The results of our microarray analysis displayed 719 gene expression differentiations (FC ≥ 2) among the analyzed 40,000 genes. The performed visualization and integrated discovery (DAVID) analysis revealed that there were interactions between various gene pathways and administration of the NPs. Based on gene ontology biological processes analysis, we found that the P53 signaling pathway, cell cycle, and cancer-affecting genes were mostly affected by the Co_2_B NPs. In conclusion, we suggested that Co_2_B NPs would be a safe and effective nanomolecule for industrial applications, particularly for medical purposes.

## 1. Introduction

In the last decade, the field of nanotechnology influenced a variety of industries enormously due to the enhanced biological and altered physical and chemical features of nanoparticles essentially affected by particle solubility, integrity, and reactivity [1]. Nanosized materials gained tremendous importance since their features increase the quality of industrial materials. Hence, different types of nanomaterial are widely used in the medical and food sectors as well as in the cosmetics field in the form of liposomes, nanosomes, inorganic compounds, dendrimers, and metals [2,3,4,5]. Considering the important and inalienable functions of nanosized materials in variegated sectors, it becomes inevitable to investigate their toxicological properties by using different approaches, including cytotoxic, genotoxic, and toxicogenomic analysis [6,7,8,9].

Cobalt borides are generally known for their reactivity and physical properties, such as biomedical, magnetic, corrosion, and hydrogen catalysis. Further, their nanosized dimensions can exhibit powerful mechanical characteristics [10]. As a class of transition metals, borides are generally recognized as very rigid materials with high-mass submodules. Critical applications of Co_2_B in a variety of reactions resulting from its functional properties gained significant attention [11]. The antioxidant effect of boron and the high electrochemical properties of cobalt makes Co_2_B a candidate catalyst for fuel cell and hydrogen storage applications [12,13]. The oxidation resistance properties of Co_2_B also make the compound a good option for surface coating applications [14]. Moreover, the magnetostrictive and magnetic properties of Co_2_B nanoparticles (NPs) also make them a good candidate for their usage in biomedical applications, including drug delivery systems and photothermal therapy [15,16,17,18].

Toxicogenomic tools allow for the revealing of the relationship between the compound–gene expression, gene expression–disease, and compound–disease and for predicting the unknown effects of chemicals on cells/tissues and different phenotypes [19]. One of the most important public toxicogenomic libraries is the Comparative Toxicogenomics Database (CTD; http://ctdbase.org; accessed on 12 March 2021), which is constructed from a wide range of data based on the interactions between genes and chemicals affecting human health [20]. The toxicogenomic approach contributes to informing people about hazardous chemicals, drugs, and stressor exposures and propounds the mechanisms underlying toxicity-related health issues. Hence, systematic analysis of the chemical structures or drugs should be performed for preparing reliable, risk-prevailing safety reports [21,22].

In this investigation, we obtained commercially available Co_2_B NPs and characterized them using X-ray crystallography (XRD), transmission electron microscope (TEM), scanning electron microscope (SEM), and energy-dispersive X-ray spectroscopy (EDX) analyses. Then, we performed a cytotoxicity evaluation on HPAEpiC cell culture for 72 h using MTT, LDH release, and NR cell viability assays. In addition, whole-genome microarray analysis was performed to identify the differentially expressed genes after treating HPAEpiC cells with Co_2_B NPs. Finally, we identified the differentially expressed genes and performed gene ontology (domain biological processes) using the database for annotation, visualization, and integrated discovery (DAVID) software and revealed the relationships between chemical–gene interactions and human diseases. The unique physicochemical properties of Co_2_B NPs make them a good candidate for use in various industries; therefore, this study was aimed to execute uncharted favorable toxicogenomic features of Co_2_B NPs for safe and serviceable utilities.

## 2. Materials and Methods

### 2.1. Nanoparticles Characterizations

Co_2_B NPs were purchased commercially (CAS No.12045-01-1, American Elements, Los Angeles, CA, USA), and the structure of the molecule was investigated via XRD, SEM, TEM, and EDX analyses. The microstructure and particle sizes of Co_2_B NPs were analyzed by a Rigaku/Smart Lab diffractometer (CuKα radiation, λ = 0.154059 nm at 40 kV and 30 mA). The measurements of the geometry were taken, coupled with θ–2θ changed between 100 and 850 with steps of 0.020. Particle size and the surface arrangement of Co_2_B NPs were investigated by a scanning electron microscope (FEI inspect S50 SEM, Thermo, Hillsboro, OR, USA) and transmission electron microscopy (JEM-ARM200CFEG UHR-TEM, JEOL, Peabody, UK). The chemical composition of Co_2_B NPs was characterized via energy-dispersive X-ray spectroscopy (EDS, EDX, Thermo, Hillsboro, OR, USA).

### 2.2. Cell Culture

Total of 10^6^ cells (HPAEpiC, ScienceCell, Carlsbad, CA, USA) were seeded in 48-well plates and incubated with Alveolar Epithelial Cell Medium (ScienceCell, USA) at 37 °C in a humidified 5% CO_2_ cell culture chamber. The experimental group consisted of 12 different concentrations of Co_2_B NPs (from ˂1 mg/L to >1000 mg/L) with hydrogen peroxide (H_2_O_2_; 25 µM Sigma-Aldrich, St. Louis, MO, USA) as positive control and negative control. Each group was conducted in triplicate to calculate standard deviations, and experiment was carried out for 72 h. To prepare Co_2_B NPs concentrations (0.625, 1.25, 2.5, 5, 10, 20, 80, 160, 320, 640, and 1280 mg/L), compounds were dispersed in <1% DMSO final concentration of medium (Sigma-Aldrich, St. Louis, MO, USA) [23].

### 2.3. MTT Assay 

A 3-(4,5-Dimethylthiazol-2-yl)-2,5-diphenyltetrazolium bromide (MTT) solution was prepared according to the manufacturer’s guide (Cayman Chemical Company, Ann Arbor, MI, USA). MTT solution was added to each experimental group and incubated for 4 h at 37 °C in a humidified 5% CO_2_ cell culture chamber. After incubation, formazan crystals were dissolved by 100 µL DMSO, and color intensity for each well was measured using a plate reader at 570 nm wavelength [24].

### 2.4. LDH Release Assay

A commercially available LDH cytotoxicity assay kit (Cayman, USA) was used to analyze cytotoxicity according to the manufacturer’s guide. Briefly, a total of 10^6^ cells were seeded in 48-well plates and exposed to Co_2_B NPs for 72 h at 37 °C in a humidified 5% CO_2_. Total of 90 µL supernatant was transferred to a new 48-well plate, and 10 µL LDH solution was added to each well. Samples were incubated at 37 °C for 30 min, and the absorbance of the samples was investigated at 490 nm using a microplate reader [25].

### 2.5. Neutral Red Assay

The neutral red assay was performed by using the In Vitro Toxicology Assay Kit (Sigma-Aldrich^®^, USA) according to manufacturer’s guide. Cell cultures were treated with the NR solution for 2.5 h at 37 °C and samples were washed with formaldehyde/CaCl_2_ solution to discard excess NR solution. Finally, the samples were incubated with acetic acid/ethanol for 30 min at room temperature to dissolve the NR solution. Samples colorimetric measurements were performed at 540 nm using a microplate reader (Bio-Tek Instruments, Winooski, VT, USA) [26].

### 2.6. Microarray Analysis

Cell cultures were exposed to IC_50_ of Co_2_B NPs in triplicate, and total RNA from the cultures was isolated by using a commercially available RNA isolation kit (Sigma-Aldrich, USA), and RNA samples purity and quantity were determined by using Agilent 2100 Bioanalyzer (Agilent Technologies, Palo Alto, CA, USA) and ND-1000 Spectrophotometer (NanoDrop, Wilmington, NC, USA). Total RNA was reverse transcribed into cDNA by using T7 oligo (dT) primers, and samples were biotinylated using TargetAmp-Nano Labeling (Lucigen, Middleton, WI, USA). Human HT-12 v4.0 Expression Beadchips (Illumina, Inc., San Diego, CA, USA) were used to hybridize cDNAs to analyze Amersham fluorolink streptavidin-Cy3 (GE Healthcare Bio-Sciences, Little Chalfont, UK) array signals under a bead array reader confocal scanner (Illumina, San Diego, CA, USA) [27].

### 2.7. Data Analysis 

All cell viability assays were analyzed statistically by using SPSS Software Version 24.0 (IBM, Armonk, NY, USA). To extract raw data from microarray analysis, Illumina GenomeStudio v2011.1 software was performed. Normalization and logarithm transformations were carried out by using the quantile method. The statistical significance of the expression data was determined as a two-fold change. Finally, the database for annotation, visualization, and integrated discovery (DAVID) analysis was used to investigate interactions between gene expression and associated biological pathways.

## 3. Results

### 3.1. Characterization of Nanoparticles 

**Figure 1:**
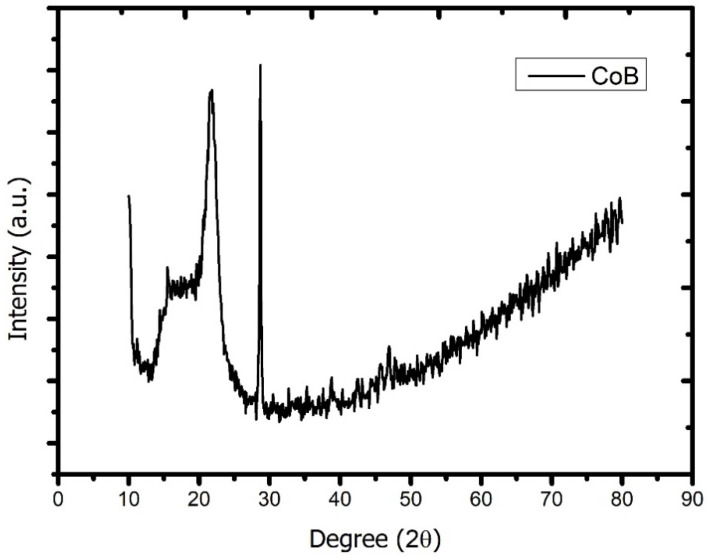
X-ray diffraction pattern of cobalt boride (Co_2_B) nanoparticles (Rigaku/Smart Lab diffractometer with CuKα radiation (λ = 0.154059 nm) operated at 40 kV and 30 mA).

We investigated the X-ray diffraction pattern of Co_2_B NPs using the Rigaku Smart Lab diffractometer with CuKα radiation (λ = 0.154059 nm) operated at 40 kV and 30 mA. According to the analysis, the dominant peak was obtained at 2θ = 29.10, and, in parallel with the literature, the investigated molecule was proven to be Co_2_B (Figure 1). In addition, the Debye Scherrer equation for the calculation of particle size is

**Figure 2:**
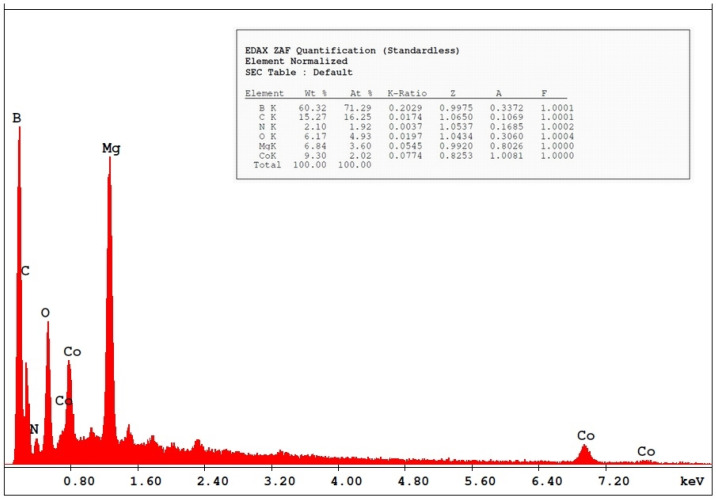
Energy-dispersive X-ray spectroscopy (EDS, EDX) results of Co2B NPs show the atomic ratios of Co/B (at %).

**Figure 3:**
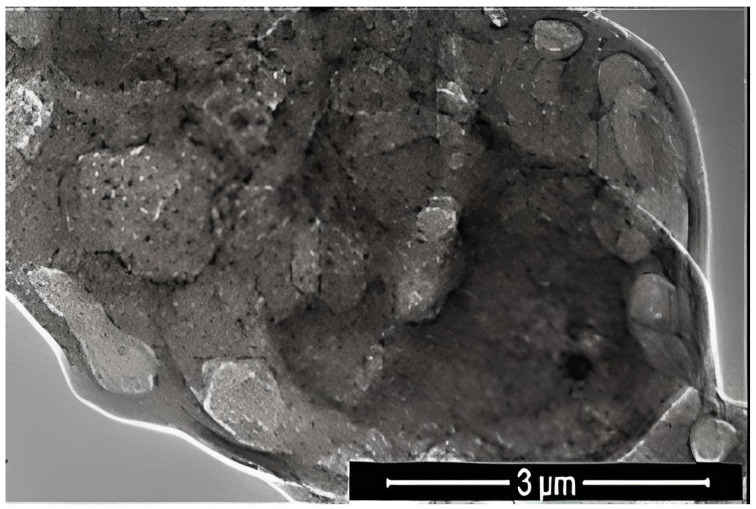
Transmission electron microscope (JEOL JEM-ARM200CFEG UHR-TEM, illustration taken as a scale of 3 µm) image of Co_2_B NPs.

**Figure 4:**
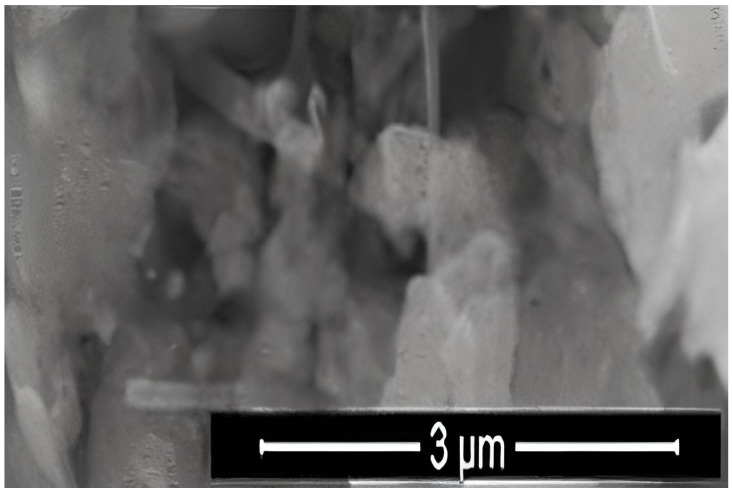
Scanning electron microscope (SEM) image (FEI inspect S50 SEM, 3 µm scale) of Co_2_B NPs.

D = Κα/βcosθ

where K is the Scherrer constant, λ is the wavelength of the X-ray beam used (1.54–184 Å), β is the full-width at half-maximum (FWHM) of the peak, and θ is the Bragg angle. The Scherrer constant denotes the shape of the particle, and its value is most commonly taken as 0.9 [X1]. The average grain size of the particles was determined as 60 nm by this equation. Energy-dispersive X-ray spectroscopy (EDS, EDX) analysis of the Co_2_B NPs was used to investigate the atomic ratios of Co/B (at %). The EDS results indicated that commercially available Co_2_B NPs were found to consist of boride and cobalt molecules and small quantities of other molecules, including carbon, oxygen, and nitrogen. On the other hand, oxygen and nitrogen measurements were probably coming from the air, and these peaks were thought to be background noises (Figure 2). The transmission electron microscope (JEOL JEM-ARM200CFEG UHR-TEM, illustration taken as a scale of 3 µm) image of Co_2_B NPs put forth that the nanoparticle size was investigated as 60 nm (Figure 3). Moreover, and scanning electron microscope (SEM) image (FEI inspect S50 SEM, 3 µm scale) of NPs (Figure 4) showed a homogeneous scattering of the Co_2_B NPs, and differential sizes ranged from 1 µm to 30 nm (Figure 4). Moreover, Zeta potential analyses put forth that the polydispersity index (PDI) of synthesized Co_2_B NPs was 4.3, which is a very high number. This result showed that Co_2_B NPs have high heterogenicity with a wide particle size range.

### 3.2. Cell Viability Analyses

**Figure 5:**
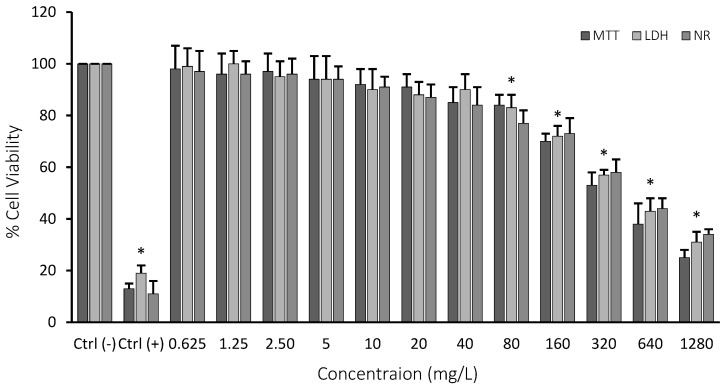
MTT, LDH, and NR assay results of cobalt boride (Co_2_B) nanoparticles on the human lung alveolar epithelial cell line. Ctrl (−): negative control (only cell culture), Ctrl (+): positive control (hydrogen peroxide (H_2_O_2_)). Symbol (*) represents statistically significant decrease in cell viability at 80 mg/L concentration, *p* < 0.05 (Microsoft Excel 2010, ANOVA: Single Factor and Regression Analysis were used to calculate the values).

All of the performed cell viability assays (MTT, LDH, and NR) indicated clear concentration-dependent cytotoxicity. The first observable toxicity (10% cytotoxicity) was monitored at 80 mg/L of Co_2_B NP concentration. IC_50_ concentration for the nanoparticle was calculated as 310.353 mg/L using single factor and regression analysis. We observed that the concentration of Co_2_B NPs should be higher than 310.353 mg/L to inhibit 50% of the growth of the HPAEpiC cell line (Figure 5). The IC_50_ concentration was used in further experimental methods in gene expression and pathway analysis.

### 3.3. Gene Expression and Pathway Analysis

**Figure 6:**
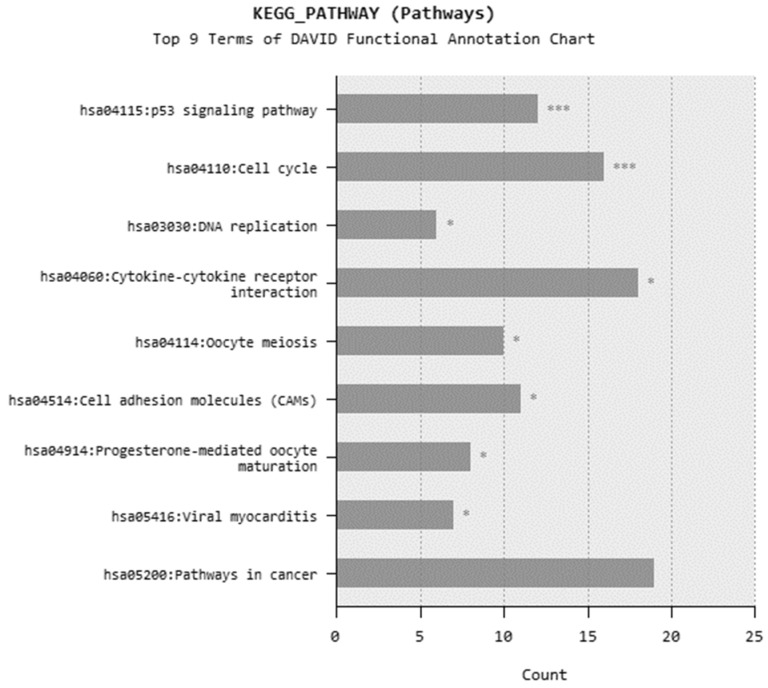
Most common KEGG pathways in DAVID functional annotation after cobalt boride (Co_2_B) NP exposure. *p*-value < 0.05 (*) and *p*-value < 0.001 (***).

**Figure 7:**
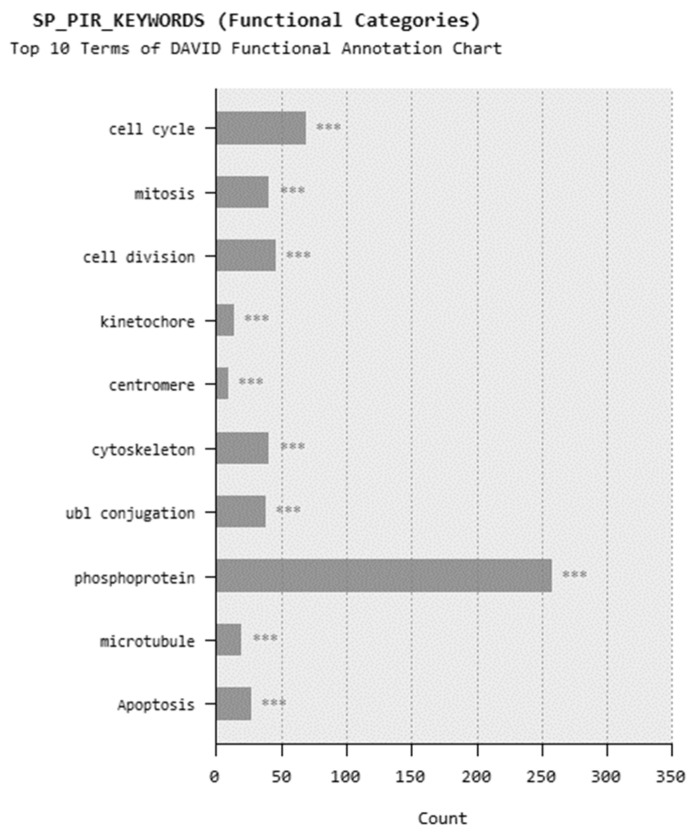
DAVID functional annotation analysis for cobalt boride (Co_2_B) NP application for functional categories. *p*-value < 0.001 (***).

**Figure 8:**
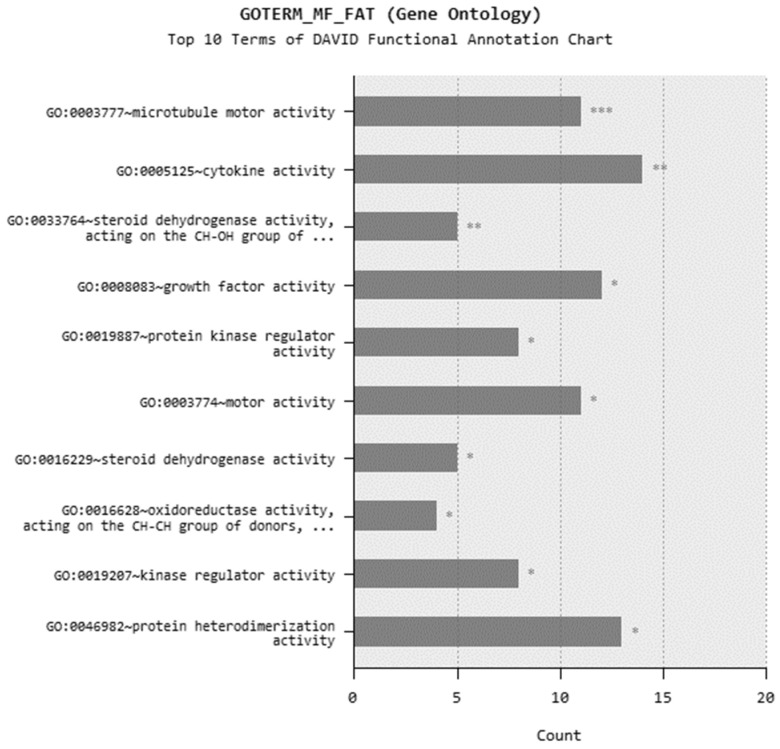
DAVID functional annotation analysis for cobalt boride (Co_2_B) NP application in gene ontology option. *p*-value < 0.05 (*), *p*-value < 0.01 (**), and *p*-value < 0.001 (***).

In microarray analysis, nine reads were performed, and the *p*-value was calculated as 0.03 for the samples. It was found that the expression of 719 genes of a total of 40,000 gene probes was significantly downregulated or upregulated (fold change ≥2) after the exposure of 310.353 mg/L Co_2_B NPs to the HPAEpiC cell culture. We found that the expression levels of 347 and 372 genes were significantly increased and decreased, respectively. Gene expression analyses showed that the five most upregulated genes were metallothionein 3 (MT3), 5S ribosomal 9 (RN5S9), eukaryotic translation elongation factor 1 alpha 2 (EEF1A2), BEN domain containing 5 (BEND5), and dihydropyrimidinase like 4 (DPYSL4) genes with fold changes (FC) of 23.08, 18.40, 12.93, 11.26, and 8.65, respectively. Moreover, it was found that the most downregulated genes were kinesin family member 20A (KIF20A), quinolinate phosphoribosyltransferase (QPRT), caspase recruitment domain family member 16 (CARD16), hyaluronan mediated motility receptor (HMMR) and RAS like family 12 (RASL12) genes with fold changes (FC) of −7.81, −5.85, −5.68, −5.22 and −4.96, respectively. The top upregulated and downregulated 25 genes were summarized in Table 1. As a result of the KEGG (Kyoto Encyclopedia of Genes and Genomes) pathway analysis using DAVID, Co_2_B was found to be more effective on the p53 signaling pathway, cell cycle, and cancer pathways (Figure 6). When DAVID functional annotation analysis for functional categories of Co_2_B NP application was evaluated, we observed that the formation of phosphoprotein structures related to the energy metabolism was significantly altered as a result of Co_2_B exposure (Figure 7). Moreover, DAVID gene ontology results revealed a significant difference associated with the in-microtubule motor protein, growth factor, and kinase protein genes in Co_2_B exposed cells (Figure 8). Microarray data were deposited in the ArrayExpress (EMBL-EBI) public database with the accession numbers E-MTAB-9035.

## 4. Discussion

In this study, Co_2_B NPs were commercially obtained, and particle characteristics were evaluated via SEM, TEM, XRD, and EDS analyses. The investigations executed concluded that Co_2_B NPs had a size of around 60 nm and a homogenous structure. With the help of XRD and EDS analysis, Co_2_B NPs contents were determined as boron and cobalt, and the molecular formulation was identified as Co_2_B. Cytotoxicity tests were performed to find out whether the Co_2_B NPs were toxic in the HPAEpiC cell line. By performing three different cytotoxicity/viability assays, it became possible to obtain more reliable results, and according to the investigations, our toxicity tests correlated with each other. Toxicity analysis showed that Co_2_B NPs should have a higher concentration to induce cytotoxicity on HPAEpiC cell culture. According to the cell viability assays, up to an 80 mg/L concentration of Co_2_B NPs did not stimulate any significant toxicity in the cell cultures. Moreover, 310.353 mg/L of Co_2_B NPs was required to complement the IC_50_ value, which was 50% of the inhibitory concentration to investigate gene expression modification for pathway analysis. Recent studies claimed that different boron compounds were investigated to have biocompatible properties in various applications. For instance, boron nitride, tungsten boride, and boron carbide nanoparticles were investigated to have lower cytotoxicity in vitro. Moreover, higher concentrations were needed to achieve significant toxicity in particular cell types [6,27,28,29]. Additionally, a study on boron nitride (BN) as a drug delivery system showed that application with 100 mg/mL of BN resulted in nearly 30% cytotoxicity on the differentiated neuron-like cells after 24 h of incubation [30]. From this result, researchers claimed that BN had low toxicity properties, and the compound was suitable for a drug delivery system. Compared to our results, an 80 mg/mL concentration of Co_2_B NPs resulted in nearly 20% cytotoxicity, which was a much lower toxic property than the previous study [31]. Moreover, chitosan nanoparticles that have been proposed as an effective drug delivery system were claimed to exhibit cytotoxic properties at the concentration of 2 mg/mL against the conjunctival cell line [32]. Furthermore, in our recent study, folic acid-conjugated boron nitride nanoparticles (FA-BN NPs) were proposed as an effective and self-assembling drug delivery system for Alzheimer’s disease (AD) treatment [7]. In the study, boron lipoic acid (BLA)- and memantine (MEM)-loaded FA-BN NPs showed promising drug delivery and release potential in the experimental AD models. 

On the other hand, there is no information about the toxicity or biological activity of Co_2_B. Thus, it was inevitable to constitute a comprehensive toxicogenomic study to understand the biological properties of Co_2_B NPs. 

Illumina microarray gene expression analysis of a total of 40,000 probes exhibited that 347 genes were found to be upregulated and 372 genes were found to be downregulated. The five most upregulated gene probes resulting from Co2B NP application could be listed as *MT3*, *EEF1A2*, *BEND5*, *DPYSL4*, and *RRAGD* genes. The *MT3* gene, one of the genes affected by cobalt boride exposure, was claimed to have proliferative, cell cycle, and apoptotic effects on cancer cells by regulating the expression of *MMP3* in the triple-negative breast cancer (TNBC) cell line [33]. Previous studies investigated that *eEF1A2* (eukaryotic protein elongation factor 1 alpha 2) was identified as a protein translation factor that has a high expression in tumors of the ovary, breast, and lung. Moreover, it was shown that *eEF1A2* could stimulate Akt and activate Akt-dependent invasion, actin remodeling, and cell migration [34,35,36]. One of the most overexpressed gene probes against Co_2_B NP application, *BEND5*, was proposed to have DNA-binding and transcription repression activities. Moreover, the hypermethylation of *BEND5* was analyzed to contribute to cell proliferation, and it was proposed as a prognostic marker for colorectal cancer [37,38]. On the other hand, the *DPYSL4* gene, shown to be overexpressed in our study, was investigated to play a crucial role in cancer suppression properties through P53 regulation and activates energy metabolism via constituting mitochondrial super-complexes. Furthermore, another study suggested that the *DPYSL4* gene could regulate epithelial cell polarization, cell proliferation, and differentiation in tooth germ morphogenesis. Moreover, it was found that the *DPYSL4* gene was a direct target for the P53 protein [39,40]. Studies showed that the Rag GTPases (*RRAGD*) could regulate the mTORC1 signaling pathway by regulating the translocation of mTORC1 to the site of activation (lysosomal surface) and result in suppression of the folliculin tumor [41,42]. According to the most upregulated genes, it was analyzed that some of them were related to carcinogenicity, invasion, and migration. On the other hand, interestingly, others were found to be related to tumor suppression and energy metabolisms.

Contrarily, when the lowest downregulated five gene probes were analyzed, it was observed that *KIF20A*, *QPRT*, *CARD16*, *HMMR*, and *RASL12* had the top place for the gene list. Firstly, the *KIF20A* gene probe was found to have the top spot for downregulated genes after Co_2_B NPs, and according to the recent analysis, the *KIF20A* gene was found to have a metastatic and proliferative impact on bladder cancer in vitro. Moreover, overexpression of the gene was investigated to prevent apoptosis and enhance cell proliferation directly in the lung adenocarcinoma cell line [43,44]. The quinolinate phosphoribosyltransferase (*QPRT*) gene was shown to be activated by Wilms’ tumor gene 1 (*WT1*) resulting in expanded antiapoptotic properties in acute myeloid leukemia and acting as an oncogenic protein. In addition, other researchers claimed that QPRT protein was observed in follicular thyroid carcinomas by immunohistochemistry techniques with a high ratio, and it could be potentially used as a new marker for follicular thyroid nodule investigations [45,46]. Another downregulated gene against Co_2_B NP exposure was *CARD16*, which was analyzed to assemble with caspase recruitment domain (CARD)-mediated caspase-1 (CASP1) and initiate inflammation through activation of interleukin (IL)-1β release [47]. Furthermore, increased expression activity of the hyaluronan-mediated motility receptor (*HMMR*) gene was reported to augment poor prognosis, aggressive phenotype, and disease progression in ovarian cancer [48]. Finally, antiapoptotic features of the *RASL12* gene were identified in a study where the cellosaurus (ANBL-6) cell line was transfected with the *ras12* gene, resulting in the prevention of doxorubicin-induced apoptosis [49]. As seen from these data from the literature, the highest downregulated genes are mainly related to cell proliferation and antiapoptotic and metastatic properties. In light of this information, it could be concluded that Co_2_B NP application inhibited gene products that enhance carcinogenic features, and the NPs contributed antitumoral formations. 

Moreover, when expressional differential top genes were investigated, it was found that the most upregulated genes, such as the *DPYSL4* and *RRAGD* genes, related to anticarcinogenic and apoptotic mechanisms. In parallel with overexpressed genes, the most downregulated genes, such as the *KIF20A*, *QPRT*, *HMMR*, and *RASL12* genes, related to tumorigenesis and antiapoptotic phenotypes. It could be understood that the anticarcinogenic upregulated genes and antiapoptotic downregulated gene sets correlated with each other. It might be comprehended from these results that Co_2_B NPs could act as an anticarcinogenic agent on the HPAEpiC cell line.

## 5. Conclusions

As a result of the DAVID analysis of KEGG pathways associated with the significantly differentiated genes, Co_2_B NPs were found to be more effective on the p53 signaling pathway, cell cycle, and cancer pathways. When the functional categories in the cells were evaluated, it was observed that the formation of the phosphoprotein structures was significantly altered as a result of the cobalt boride application. In addition, DAVID gene ontology results revealed a significant change in microtubule motor protein, growth factor, and kinase protein expression in Co_2_B NPs exposed to HPAEpiC cells. According to the cytotoxicity and gene expression analyses, it could be concluded that industrial application of Co_2_B NPs would not cause serious toxicological outcomes if the NPs concentrations reach higher rates. Co_2_B NPs exhibited good potency for advanced uses, especially in biomedical applications, due to their positive impact on global gene expression profiling. On the other hand, our preclinical data put forth highly safe properties of the NPs, and comprehensive in vivo investigations should be performed to constitute a more confident safety report for Co_2_B NP applications. Moreover, different polymeric nanoparticle structures should be investigated to obtain more comprehensive analyses to conclude if different Co_2_B architectures have similar results. 

## Figures and Tables

**Table 1:** A total of 25 upregulated and downregulated genes subject to cobalt boride (Co_2_B) NP application.

Cobalt Boride (Co_2_B) Fold Change (FC)			
Upregulated Genes	FC	Downregulated Genes	FC
MT3	23.08	KIF20A	−7.81
RN5S9	18.40	QPRT	−5.85
EEF1A2	12.93	CARD16	−5.68
BEND5	11.26	HMMR	−5.22
DPYSL4	8.65	RASL12	−4.96
RRAGD	8.12	VWA5A	−4.94
RNASE4	7.90	DLGAP5	−4.92
GALNTL4	7.67	TNFSF11	−4.72
CYP3A7	6.95	LOC100134259	−4.68
BTG2	6.76	CENPA	−4.52
TMEM145	6.74	TOP2A	−4.46
NDUFA4L2	6.68	DLGAP5	−4.45
RRAD	6.43	CXCL12	−4.44
RNASE4	6.31	ECHDC2	−4.44
HIST1H2BD	6.27	CASP1	−4.43
TNFSF13B	6.21	HMMR	−4.36
VLDLR	6.17	TNFRSF11B	−4.31
HES4	6.05	CD248	−4.24
IGFBP3	5.84	CDC20	−4.21
RRAD	5.74	CCNB2	−4.05
GDF15	5.65	CCL2	−4.02
ANGPTL4	5.63	GSTM5	−4.00
IGFBP3	5.50	FAM83D	−3.97
BHLHB3	5.31	SCG5	−3.95
ATF3	5.27	ASPM	−3.83

## Data Availability

The data that support the findings of this study are openly available in ArrayExpress at https://www.ebi.ac.uk/biostudies/arrayexpress/studies/E-MTAB-9035; accessed on 15 March 2021, reference number E-MTAB-9035.
